# Financial Hardship and the Pain of Social Disconnection During the COVID-19 Pandemic in the United States

**DOI:** 10.1093/geroni/igab046.362

**Published:** 2021-12-17

**Authors:** Tirth Bhatta, Nirmala Lekhak, Timothy Goler, Eva Kahana, Sfurti Maheshwari

**Affiliations:** 1 University of Nevada, Las Vegas, Nevada, United States; 2 University of Nevada Las Vegas, Las vegas, Nevada, United States; 3 Norfolk State University, Newport News, Virginia, United States; 4 Case Western University, Case Western reserve University, Ohio, United States; 5 University of Nevada, Las Vegas, Las Vegas, Nevada, United States

## Abstract

Considerable scholarly attention has been directed at increasing social isolation and loneliness during the COVID-19 pandemic, and their adverse impact on later life psychological well-being. Notably absent is the focus on financial hardship in the context of overlapping unprecedented economic and public health crisis. It is unclear whether loneliness continues to differ across different levels of financial hardship even amidst immense uncertainty, social isolation, and anxiety induced by the pandemic. Based on our nationwide web-based survey of adults aged 50 years and older (n=1861), we used ordinal logistic regression to examine the influence of financial hardship on loneliness and assessed the role of socioeconomic status (SES), emotional support, and health status in contributing to such influence. We found significantly higher odds of greater loneliness (β = .28, p < .001) among individuals who reported experiencing greater financial hardship. Among two measures of SES, only household income contributed substantially to the influence of financial hardship on loneliness. We documented significantly lower emotional support and greater health disadvantage among individuals experiencing greater financial hardship. Consideration of emotional support and health status explained the remaining influence of financial hardship, due to their association with both financial hardship and loneliness. Despite a sense of shared vulnerability and social isolation across the general population, our findings suggest that SES inequalities in later life loneliness are maintained even in the midst of the pandemic.

